# Effects of active, inactive, and derivatives of *Akkermansia muciniphila* on the expression of the endocannabinoid system and *PPARs* genes

**DOI:** 10.1038/s41598-022-13840-8

**Published:** 2022-06-15

**Authors:** Farinaz Ghaderi, Fattah Sotoodehnejadnematalahi, Zahra Hajebrahimi, Abolfazl Fateh, Seyed Davar Siadat

**Affiliations:** 1 Department of Biology, Science and Research Branch, Islamic Azad University, Tehran, Iran; 2 A&S Research Institute, Ministry of Science Research and Technology, Tehran, Iran; 3 Department of Mycobacteriology and Pulmonary Research and Microbiology Research Center (MRC), Pasteur Institute of Iran, Tehran, Iran; 4 Microbiology Research Center (MRC), Pasteur Institute of Iran, Tehran, Iran

**Keywords:** Extracellular signalling molecules, Microbiology, Microbial communities

## Abstract

This study aimed to investigate the effects of active and heat-inactivated forms of *Akkermansia muciniphila*, bacterium-derived outer membrane vesicles (OMVs), and cell-free supernatant on the transcription of endocannabinoid system (ECS) members, including cannabinoid receptors 1 and 2 (CB1 and CB2), fatty acid amide hydrolase (FAAH), and peroxisome proliferator-activated receptors (PPARs) genes (i.e., *α, β/δ, and δ*) in Caco-2 and HepG-2 cell lines. After the inoculation of *A. muciniphila* in brain heart infusion enriched medium, OMVs and cell-free supernatant were extracted. For the investigation of the effects of bacteria and its derivatives on the expression of ECS and *PPARs* genes, the aforementioned cells were treated by active and heat-inactivated bacteria, OMVs, and cell-free supernatant. Quantitative real-time polymerase chain reaction analysis revealed that both forms of the bacterium, bacterial-derived OMVs, and cell-free supernatant could affect the expression of *CB1*, *CB2*, *FAAH*, and *PPARs* genes (i.e., *α, β/δ, and δ*) significantly (*P* < 0.05). Considering the engagement of the aforementioned genes in metabolic pathways, it might be suggested that both forms of the bacterium, OMVs, and cell-free supernatant might have the potential to serve as a probiotic, paraprobiotic, and postbiotic candidate to prevent obesity, metabolic disorders, and liver diseases.

## Introduction

One of the most remarkable habitats for microbiota is the human gastrointestinal tract (GIT), where variable microbes colonize from the mouth down to the rectum according to environmental conditions^1,2^. Gut microbiota executes several structural, protective, and metabolic functions and roles, such as interfering in gut barrier functions, insuring its homeostasis, supplying nutrients, participating in signaling network, regulating epithelial tissue development, and affecting the immune system in the intestinal mucosa^3^. The appropriate physiology and homeostasis of the host are critically associated with the integrity of the intestinal barrier^4–6^. The changes in the latter might lead to gastrointestinal^4,7^, metabolic^4,8^, and even neurological disorders^4,9^. Accordingly, leaky gut syndrome, as one of the most important gastrointestinal disorders, can occur by possible alteration in tight junction proteins. Leaky gut syndrome might cause the translocation of intestinal microbiota and their microbial-associated molecular patterns (MAMPs) toward the liver through the gut-liver axis. It is well documented that this phenomenon can induce numerous liver disturbances^10,11^.

Among diverse bacterial species in the gut, *Akkermansia. muciniphila* is an anaerobic Gram-negative and a mucus colonizing bacterium^12–15^. It appropriates about 3–5% of the gut microbiota in healthy humans^14,16^, has a major contribution in gut barrier regulation, and involves in metabolic and homeostatic procedures^4,17^. Recently, it has been considered a candidate of the next generation probiotics^12^. In recent years, it has been specified that heat-inactivated bacteria might exert some active molecules and MAMPs that influence the host physiology, immunity, and gut barrier integrity^18,19^.

Several studies have shown that various living and nonliving forms of *A. muciniphila* in inflammatory conditions and metabolic diseases have been able to prevent the onset of disease or relieve the symptoms of the disease^20–22^. Additionally, to avoid inflammatory and antibiotic resistance side effects, the administration of bacterial cell-free supernatant is suggested as a substitution for alive bacteria since it contains all the beneficial and functional metabolites of the bacterial flora^23^. Outer membrane vesicles (OMVs) in Gram-negative bacteria have shown efficient roles not only in bacterial survival but also in interacting with the host through inter- and intra-kingdom communications without actual intercellular connection^21,24^.

The evidence suggests an interaction between the endocannabinoid system (ECS) and gut microbiota and subsequently the liver through the gut-liver axis in some leaky gut conditions^25^. The ECS consists of three main compartments, including endocannabinoids (eCBs), cannabinoid receptors (i.e., CB1R and CB2R), and related metabolic enzymes^25–27^. It participates in numerous biological processes in the brain and some peripheral tissues^28^. Cannabinoid receptors are coupled with Gi/o family of the G-proteins through which involve in signal transduction pathways^29^. A neuronal stimulation leads to release of endocannabinoids from the postsynaptic terminals to the extracellular space of the synaptic terminal in a retrograde manner and binding to CB1/2 receptors in the presynaptic terminals^30^. Activity of the Biosynthetic enzymes depends on intracellular Ca^2+^ concentration which determines the synthesis of endocannabinoids^31^. Fatty acid amide hydrolase (FAAH) is located in the postsynaptic terminal to hydrolyze these ligands. Since CB1/2 inhibit Ca^2+^ influx (through regulation of ion channels), Gamma amino butyrate (GABA) and glutamate release, and also nitric oxide (NO) production, it can modulate neurotransmission, inhibit adenylate cyclase and cAMP/Protein kinaseA pathway, respectively. They can also activate mitogen-activated protein kinases (MAPKs) such as ERK1/2, P38 and JNK which depending on the type of ligand and cell environmental conditions, the result of this signaling pathway will be different^30,32^. In GIT, CB1 modulates the motility, and reduces the secretion of fluids, gastric acids, neurotransmitters, and hormones along with increasing the gut permeability^30^. High fat diets induce cholecystokinin (CCK) production by enteroendocrine l-cells that leads to induce satiety signals via afferent sensory vagus nerves to the brain. This procedure is inhibited by CB1 probably through MAPK signaling and promotes over eating followed by diet induced obesity^33,34^. Within hepatocytes, CB1 is able to increase the expression of a lipogenic transcription factor of sterol regulatory element binding protein (SREBP). Enhancement of acetyl-CoA carboxylase 1(ACC) and fatty acid synthase (FAS) as SREBP targets lead to fatty liver, hypertriglyceridemia, and insulin resistance^32,35^. CB2 signaling in hepatocytes have shown a regulatory effect on lipid accumulation^36^, decreasing the inflammatory cytokines, steatosis, hepatic proliferation, and liver fibrosis progression^32,37^. Hence, The ECS functions might lead to increased permeability, decreasing inflammation, regulating food intake, and gut motility in the GIT. It also influences some metabolic disorders, such as obesity, type 2 diabetes, steatosis, fibrogenesis, alcoholic and nonalcoholic liver diseases, mainly through CB1R function^25,26,28^.

Peroxisome Proliferator-Activated Receptors (PPARs) are nuclear receptors with the affinity to bind to endocannabinoids. They make a role in gene regulation of fatty acid oxidation, metabolism of carbohydrates and lipids, cell proliferation, and inflammation^32,38,39^. Several studies have shown considerable interactions between the gut microbiota and peroxisome proliferator-activated receptors (PPARs) that participate in the regulation of gut barrier permeability, energy homeostasis, and liver metabolism^27,40^. Since there is a cross-reaction between ECS members and PPARs genes^41^ and regarding the functions of the ECS in the gut^25,42^ and liver^43,44^, the current study aimed to evaluate the effects of active and inactive forms of *A. muciniphila*, its derived OMVs, and cell-free supernatant on the expression of remarkable genes, such as *CB1*, *CB2*, fatty acid amide hydrolase (*FAAH*) as the representatives of the ECS, and *PPARs* (i.e., *α*, *β/δ*, and *δ*) genes which are all involved in the regulation of numerous metabolic, inflammatory, and developmental processes in the gut and liver.

## Materials and methods

### Bacterial culture condition

*Akkermansia muciniphila* (ATCC BAA-835) was provided from the DSMZ institute (German Collection of Microorganisms and Cell Cultures GmbH). *A. muciniphila* strains were cultured in brain heart infusion (BHI) agar medium (Quelab, Canada) supplemented with 0.5% mucin-type III (Sigma-Aldrich, St. Louis, Missouri, USA), hemin (5 μg/ml), menadione (1 μg/ml)^45^ and 0.05% L-cysteine^46^ under anaerobic conditions (80% N_2_, 10% H_2_, and 10% CO_2_) at 37 °C for 3–7 days as described previously^12^.

Polymerase chain reaction (PCR) test was performed based on 16 s ribosomal ribonucleic acid (rRNA) sequence recognition to confirm the *A. muciniphila*, in addition to macroscopic and microscopic (Gram staining) assays (Supplementary Table 1).

Cell-free supernatant was obtained from the inoculation of *A. muciniphila* in 100 mL BHI broth with the above-mentioned supplementations incubated in the same condition reaching optical density (OD) of 1.5 in 600 nm, centrifuged in 8000 × g for 5 min; the pH was adjusted in 7.4 and then purified by passing through 2.22 nm filters and kept at −70 °C until usage.

### OMVs extraction and confirmation

12 × 10^8^ colony-forming units (CFU/ml) of bacteria equal to 4 McFarland were inoculated in above-mentioned supplemented BHI broth overnight, and when the OD reached 1.5 in 600 nm^4^, the bacterial suspension were centrifuged at 6000 × g at 4 °C. The supernatant was poured out, and the pellets were washed twice with phosphate-buffered saline (PBS) followed by centrifugation at 6000 × g at 4 °C, suspended with 9% sodium chloride solution at 6000 × g at 4 °C for an hour and then mixed with ethylenediaminetetraacetic acid-sodium deoxycholate (Sigma Aldrich, USA) buffers^47^. In advance, centrifugation was performed at 20,000 × g at 4 °C for an hour and ultracentrifugation at 125,000 × g twice for 2 h sequentially, and then it was kept in sucrose 3% at −70 °C^48^.

Sodium dodecyl sulfate–polyacrylamide gel electrophoresis (SDS-PAGE) procedure was performed for the evaluation of the protein content of the sample, and transmission electron microscopy (TEM) was performed as a confirmation for the presence and sizes of OMVs. The sample was prepared by negative staining and then observed by PHILIPS (Netherlands) EM 208.

### Cell culture

Caco-2 (IBRC C10094) and Hep-G2 (C10096) cell lines were obtained from Iranian Biological Resource Center, Tehran, Iran, cultured in Dulbecco’s Modified Eagle’s Medium (Bioidea, Iran) supplemented by 10% heat-inactivated (56 °C, 30 min), fetal bovine serum (Bioidea, Iran), 1% nonessential amino acids (Bioidea, Iran), and 1% penicillin–streptomycin (Bioidea, Iran), which were incubated at 37 °C and 5% CO_2_ condition^49,50^. After reaching enough confluency (minimum 70%) and transferring 5 × 10^5^ cells into every well of six well plates (Sorfa, China)^12^, with some modifications, compared to previous studies, four types of treatments, including active and inactivated (20 min in 56 °C) *A .municiphila*, OMVs, and cell-free supernatant, were administered on both Caco-2 and HepG-2 cells. The cells were separately infected by active and inactivated bacteria at the multiplicity of infection (MOI) both in 10, 50, and 100 ratios (i.e., 10, 50, and 100 bacteria per cell, respectively). Some other cells in other wells were treated by 50 and 100 μg/mL OMVs and others by a well-optimized concentration of 7% (v/v [medium/cell-free supernatant]) cell-free supernatant ^12,47,49,51^. Equal volumes of PBS, sucrose 3%, and supplemented BHI broth were used in separated wells as controls for comparison with their related wells. All treated cells were incubated for 24 h^12,47^.

### RNA extraction and cDNA synthesis

The cells were collected from six wells (5 × 10^5^ cells per well), and their total RNA was extracted using RNX-Plus Solution (2000 ng/μl; Sinacolon, Iran). In order to purify the extracted RNAs, they were treated by DNAse for 1 h at 37 °C. The presence and quality of the extracted RNAs were checked out by agarose gel electrophoresis and spectrophotometry using NanoDrop 2000 (Thermo Fisher Scientific, USA). After balancing the concentrations of RNAs, complementary deoxyribonucleic acid (cDNA) was synthesized using a cDNA synthesis kit (Parstous, Iran) according to manufacturers’ instructions. A no reverse transcriptase control cDNA was included in qRT-PCR analysis. The concentrations were checked out by spectrophotometry using a Nanodrop device^47^.

### Quantitative real-time PCR analysis of ECS-related (i.e., *CB1*, *CB2*, and *FAAH*) and *PPARs* genes

Quantitative real-time polymerase chain reaction (qRT-PCR) was performed using SYBR Green real-time master mix (Parstous, Iran), specific primers, and newly synthesized cDNAs in LightCycler®96 SW 1.1 instrument (Roche, Germany). Glyceraldehyde 3-phosphate dehydrogenase was used as a reference gene. The program conditions consisted 1 cycle of 94 °C for 10 min followed by 40 cycles, including denaturation at 95 °C for 15 s, annealing temperatures of each set of primers for 30 s, and 72 °C as an extension time for 30 s followed by 10 min at 72 °C for the complementation of the procedure.

### Statistical analysis

The data extracted from qRT-PCR were analyzed by GraphPad Prism software (version 8.4.3; GraphPad Software Inc., San Diego, CA, USA) using an independent sample t-test (between two groups) and one-way analysis of variance (with Tukey’s multiple comparison test to estimate differences between means which was used to compare means among more than two groups for each parameter). All results were considered statistically significant with a *p-value* less than 0.05^12,47^.

## Results

### Confirmation of *A. municiphila* and Its OMVs with size

**Figure 1:**
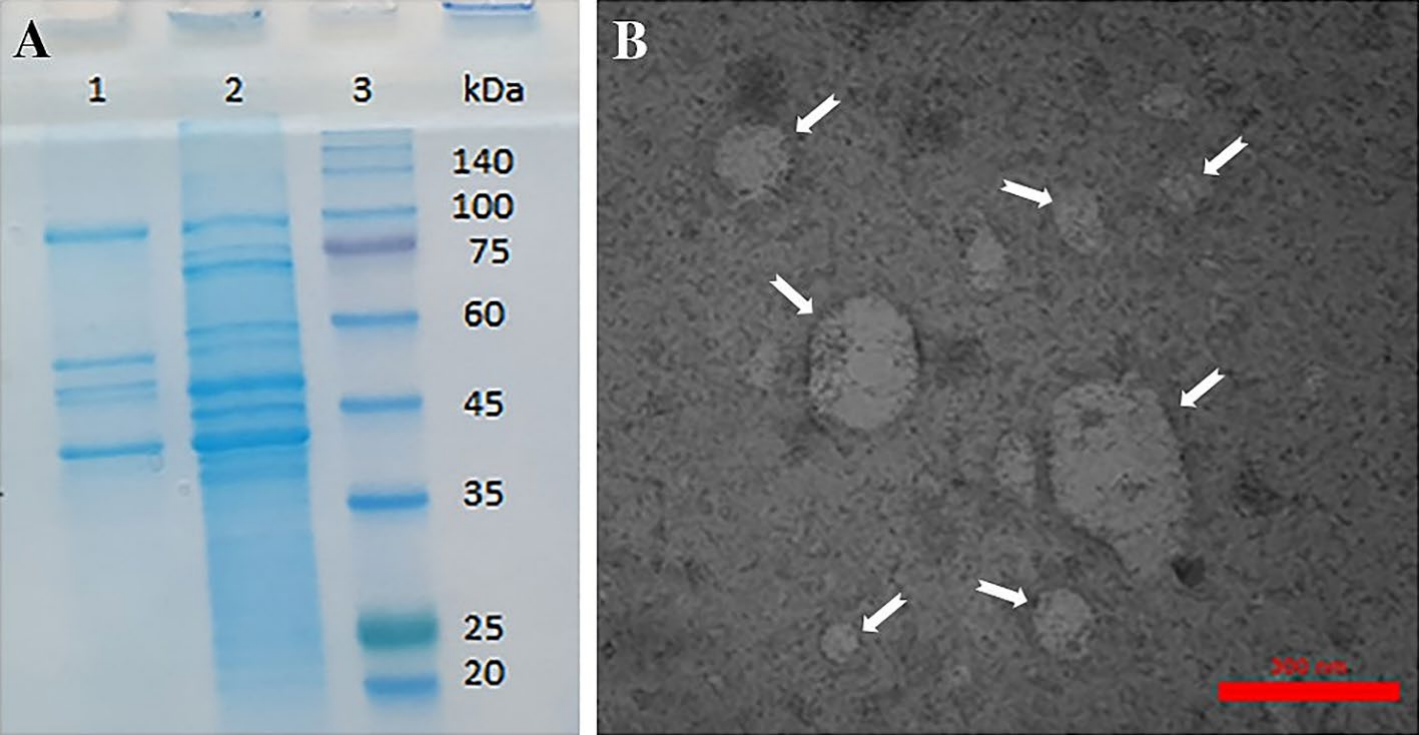
*A.muciniphila* OMVs confirmation. (**A**) SDS-PAGE. (1) OMVs proteins (2) supernatant (3) molecular weight Protein Marker (**B**) TEM image × 18,000. Arrows indicate OMVs in different sizes (30–300 nm).

*A. municiphila* was confirmed by PCR methods (*16 s rRNA* gene PCR). The SDS-PAGE displayed the protein content of the bacteria, and TEM analysis confirmed the presence of *A. muciniphila-*derived OMVs in size (30–300 nm vesicles; Fig. 1).

### qRT-PCR analysis of the associated genes expression in Caco-2 cell line

#### Effects of active and inactivated *A. muciniphila*, derived OMVs, and cell-free supernatant on transcription level of the studied genes Involved in endocannabinoid system

**Figure 2:**
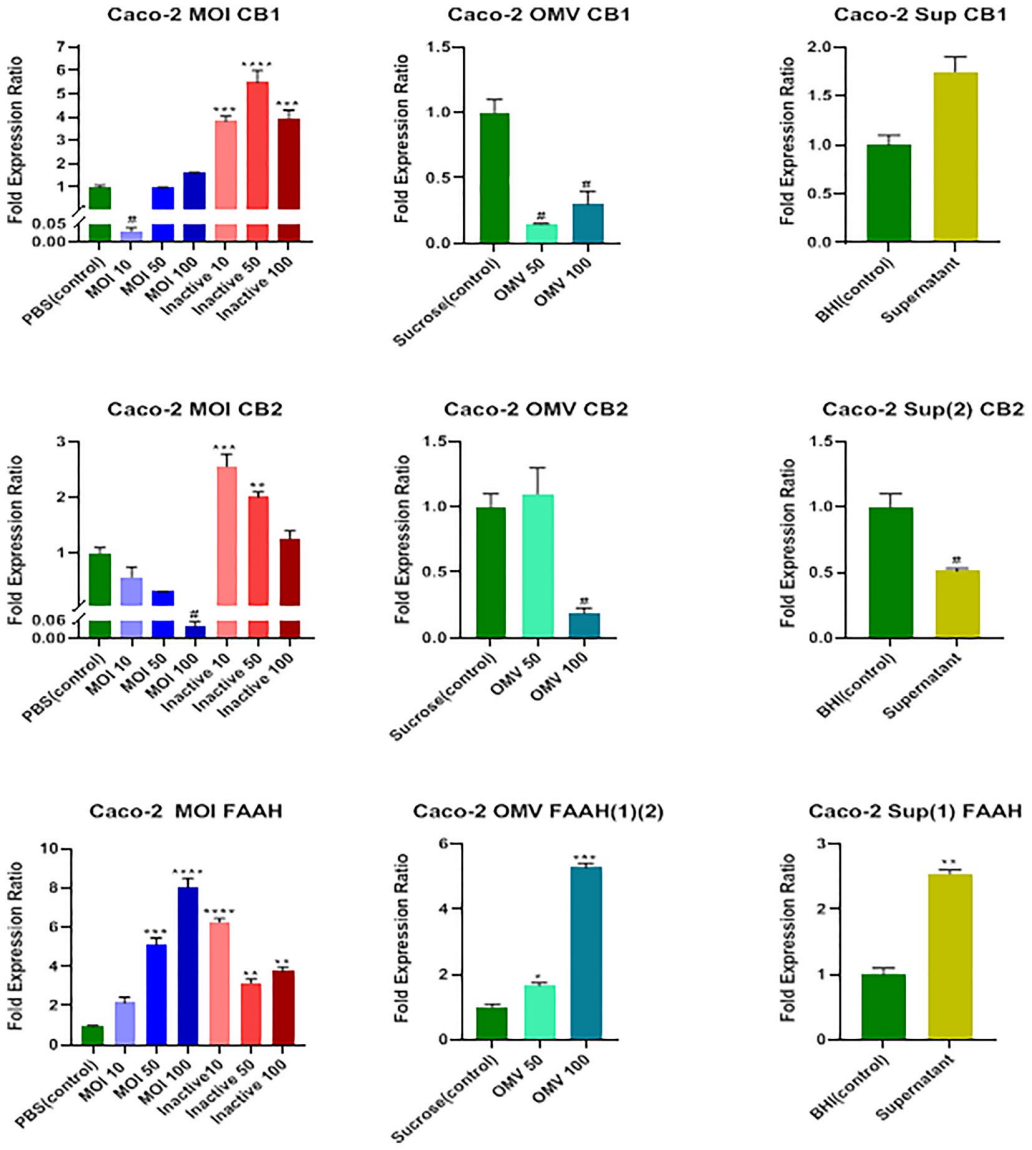
Effects of active and inactive *A.muciniphila* (at MOIs of 10, 50 and 100), its derived OMVs (50 and 100 μg/mL), and 7% cell free supernatant on the expression of endocannabinoid system related genes (CB1, CB2 receptors, and FAAH) in Caco-2 cells. Significancy is evaluated in comparison with control. Data are shown as the mean ± SEM. (*) and (^#^) represent significant increase and decrease, respectively.*/^#^ *P* < 0.05, **P < 0.01, ***P < 0.001, and ****P < 0.0001 by one-way ANOVA and t-test statistical analysis. MOI: multiplicity of infection, OMV: outer membrane vesicle, Sup: supernatant.

Data analysis showed a significant decrease in the messenger ribonucleic acid (mRNA) level of the CB1 receptor by MOI 10 of active *A. muciniphila* (*P* = 0.04) and both 50 and 100 μg/mL concentrations of OMVs (*P* = 0.01) (Fig. 2). All three MOIs of 10, 50, and 100 of the inactivated form of the bacterium could remarkably (*P* = 0.0008, *P* = 0.0001*,* and *P* = 0.0006, respectively) increase the mRNA level of the CB1 receptor; nevertheless, the cell-free supernatant did not have a significant effect on the expression of the *CB1* gene (*P* = 0.053).

A noticeable decrease occurred in the mRNA level of the CB2 receptor by MOI 100 of active *A. muciniphila*, 100 μg/ml concentrations of OMV, and concentration of 7% (v/v [medium/cell-free supernatant]) cell-free supernatant (*P* = 0.01, *P* = 0.03, and *P* = 0.04, respectively); however, the MOIs of 10 and 50 of the inactivated form of the bacterium increased the mRNA level of the CB2 receptor significantly (*P* = 0.0007 and *P* = 0.01, respectively) (Fig. 2).

As shown in Fig. 2, MOIs 50 and 100 of active *A .muciniphila* (*P* = 0.0002 and *P* < 0.0001, respectively), all three MOIs of 10, 50, and 100 of the inactivated form of the bacterium (*P* < 0.0001, *P* = 0.0075, and *P* = 0.0018, respectively), both 50 and 100 μg/ml concentrations of OMVs (*P* = 0.033 *and P* = 0.0002, respectively), and 7% (v/v [medium/cell-free supernatant]) concentration of cell-free supernatant (*P* = 0.0057) increased the mRNA level of the *FAAH* gene remarkably.

#### Effects of the above-mentioned treatments on transcription level of *PPARs *(i.e., *α*, *β/δ*, and *ϒ*) genes

**Figure 3:**
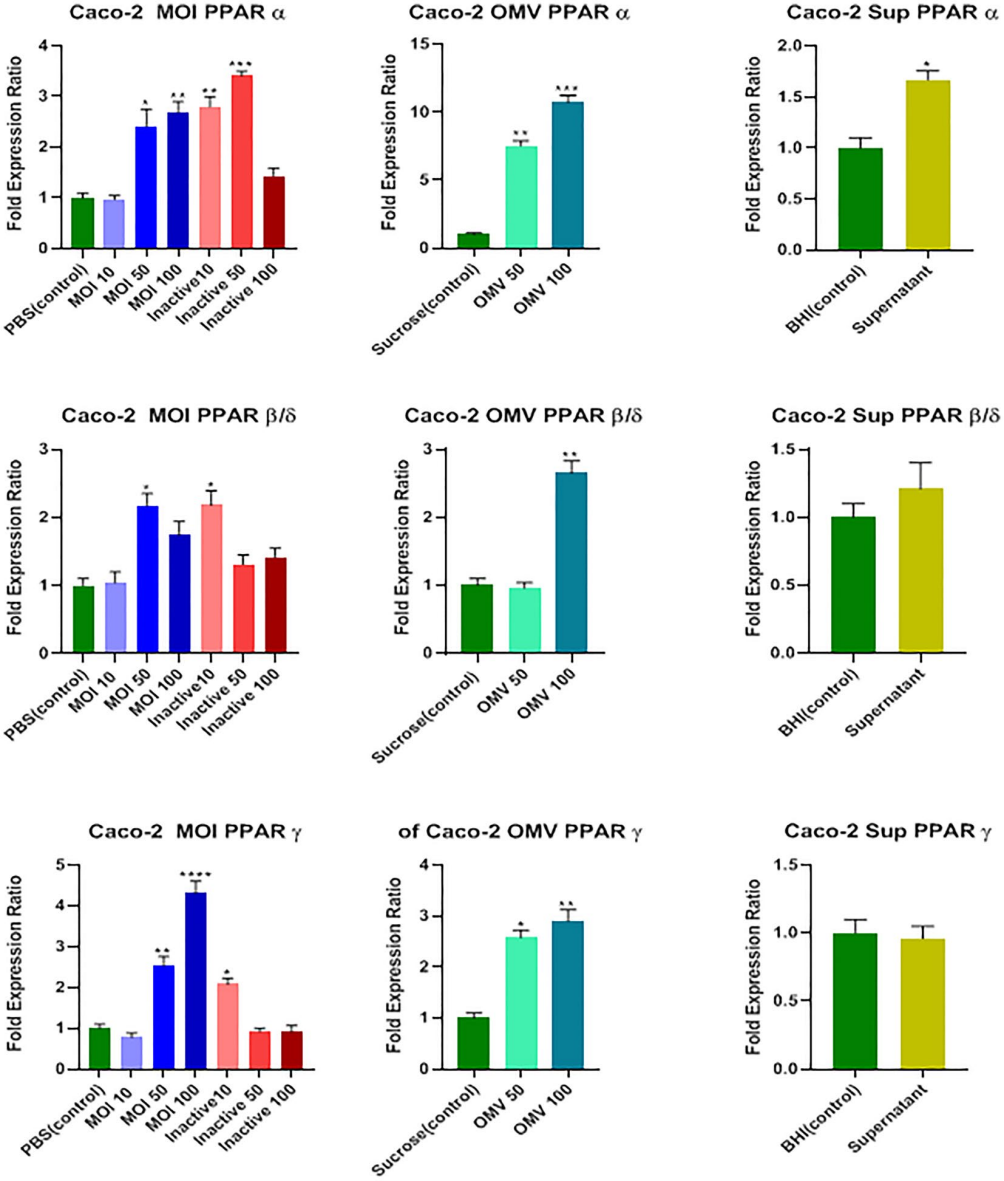
Effects of active and inactive *A.muciniphila* (at MOIs of 10, 50, and 100), its derived OMVs (50 and 100 μg/mL) and 7% (v/v [medium/cell-free supernatant]) cell free supernatant on the expression of PPARS (α, β/δ, and ϒ) genes in Caco-2 cells. Significancy is evaluated in comparison with control. Data are shown as the mean ± SEM. (*) and (^#^) represent significant increase and decrease, respectively.*P < 0.05, **P < 0.01, ***P < 0.001, and ****P < 0.0001 by one-way ANOVA and t-test statistical analysis. MOI: multiplicity of infection, OMV: outer membrane vesicle, Sup: supernatant.

In reference to Fig. 3, MOIs 50 and 100 of active *A. muciniphila* (*P* = 0.011 and *P* = 0.004, respectively), MOIs of 10 and 50 of the inactivated form of the bacterium (*P* = 0.002 and *P* = 0.0004, respectively), both 50 and 100 μg/ml concentrations of OMVs (*P* = 0.002 and *P* = 0.0008, respectively), and 7% (v/v [medium/cell-free supernatant]) concentration of cell-free supernatant (*P* = 0.038) could significantly increase the mRNA level of the *PPARα* gene.

The MOI 50 of active *A. muciniphila*, MOI 10 of the inactivated form of the bacterium, and 100 μg/ml concentration of OMV increased the mRNA level of the *PPARβ/δ* gene remarkably (*P* = 0.015, *P* = 0.014, and *P* = 0.005, respectively*)*; nonetheless, the cell-free supernatant could not affect *PPARβ/δ* transcriptome level (*P* = 0.43; Fig. 3).

The MOIs 50 and 100 of active *A. muciniphila* (*P* = 0.004 and *P* < 0.0001, respectively), MOI 10 of the inactivated form of the bacterium (*P* = 0.03), and both 50 and 100 μg/mL concentrations of OMVs (*P* = 0.012 and *P* = 0.007, respectively) significantly increased the mRNA level of *PPARϒ* gene; nevertheless, the cell-free supernatant could not affect its expression significantly (*P* = 0.79; Fig. 3).

### qRT-PCR analysis of the associated genes expression in HepG-2 cell line

#### Effects of active and inactivated *A. muciniphila*, derived OMVs, and cell-free supernatant on transcription level of the studied genes involved in endocannabinoid system

**Figure 4:**
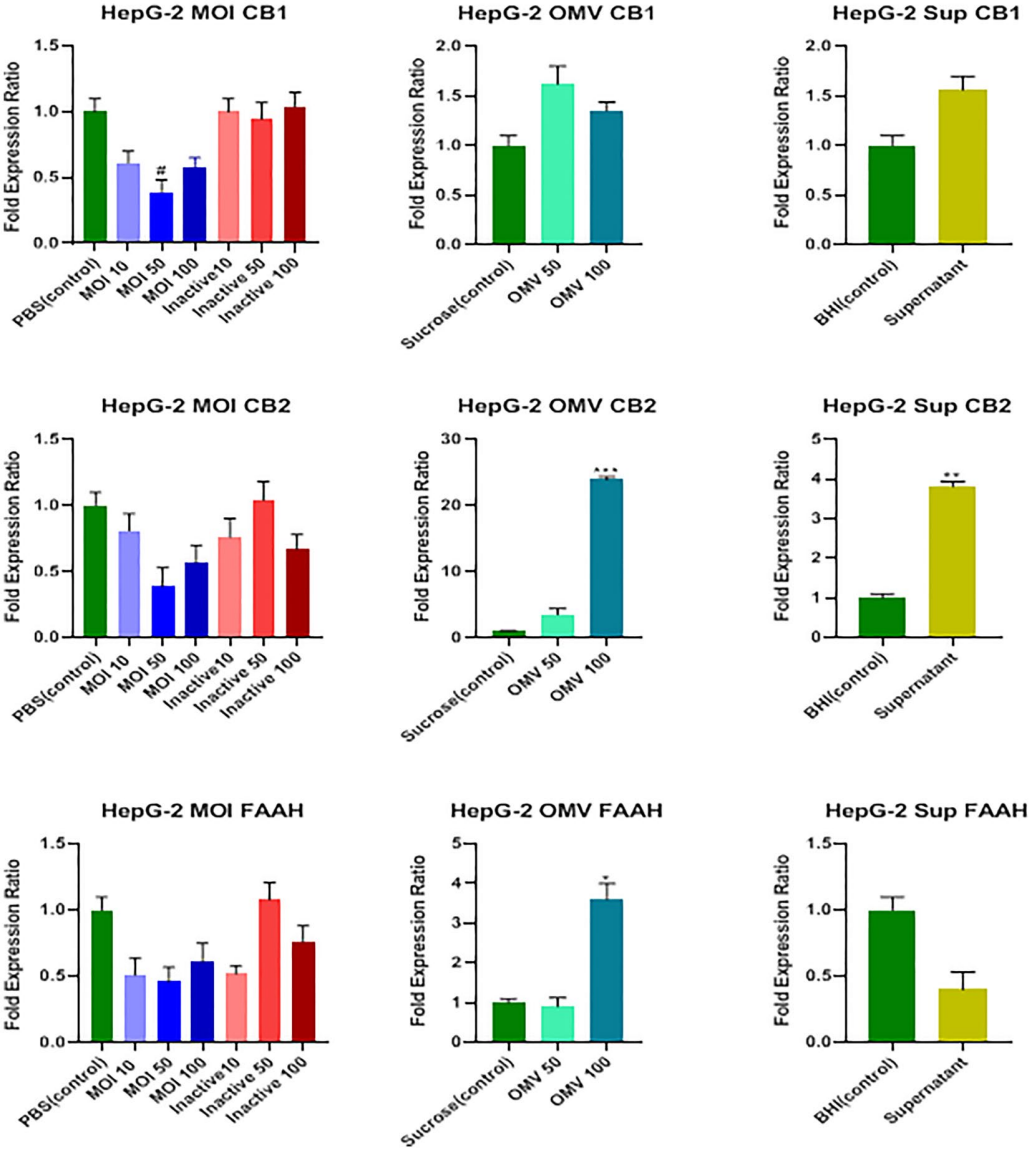
Effects of active and inactive *A.muciniphila* (at MOIs of 10, 50, and 100), its derived OMVs (50 and 100 μg/mL), and 7% (v/v [medium/cell-free supernatant]) cell free supernatant on the expression of endocannabinoid system related genes (CB1, CB2 receptors, and FAAH) in HepG-2 cells. Significancy is evaluated in comparison with control. Data are shown as the mean ± SEM. (*) and (^#^) represent significant increase and decrease, respectively.*P < 0.05, **P < 0.01, ***P < 0.001 by one-way ANOVA and t-test statistical analysis. MOI: multiplicity of infection, OMV: outer membrane vesicle, Sup: supernatant.

Data analysis showed that only MOI 50 of the active form of the bacterium significantly decreased the CB1 receptor transcription level (*P* = 0.033), and none of the inactive bacteria, OMVs, and cell-free supernatant could affect the transcription level of the CB1 receptor (*P* > 0.05; Fig. 4).

According to the results shown in Fig. 4, 100 μg/mL concentrations of OMVs and 7% (v/v [medium/cell-free supernatant]) concentration of cell-free supernatant remarkably increased the mRNA level of the CB2 receptor (*P* = 0.0002 and *P* = 0.0034, respectively); nonetheless, neither active nor inactive forms of the bacterium affected its transcription level (*P* > 0.05). Only 100 μg/mL concentrations of OMVs increased *FAAH* mRNA level significantly (*P* = 0.013), and none of the concentrations of the active and inactive forms and cell-free supernatant affected it (*P* > 0.05; Fig. 4).

#### Effects of the above-mentioned treatments on transcription level of *PPARs* (i.e., *α, β/δ, and ϒ*) genes

**Figure 5:**
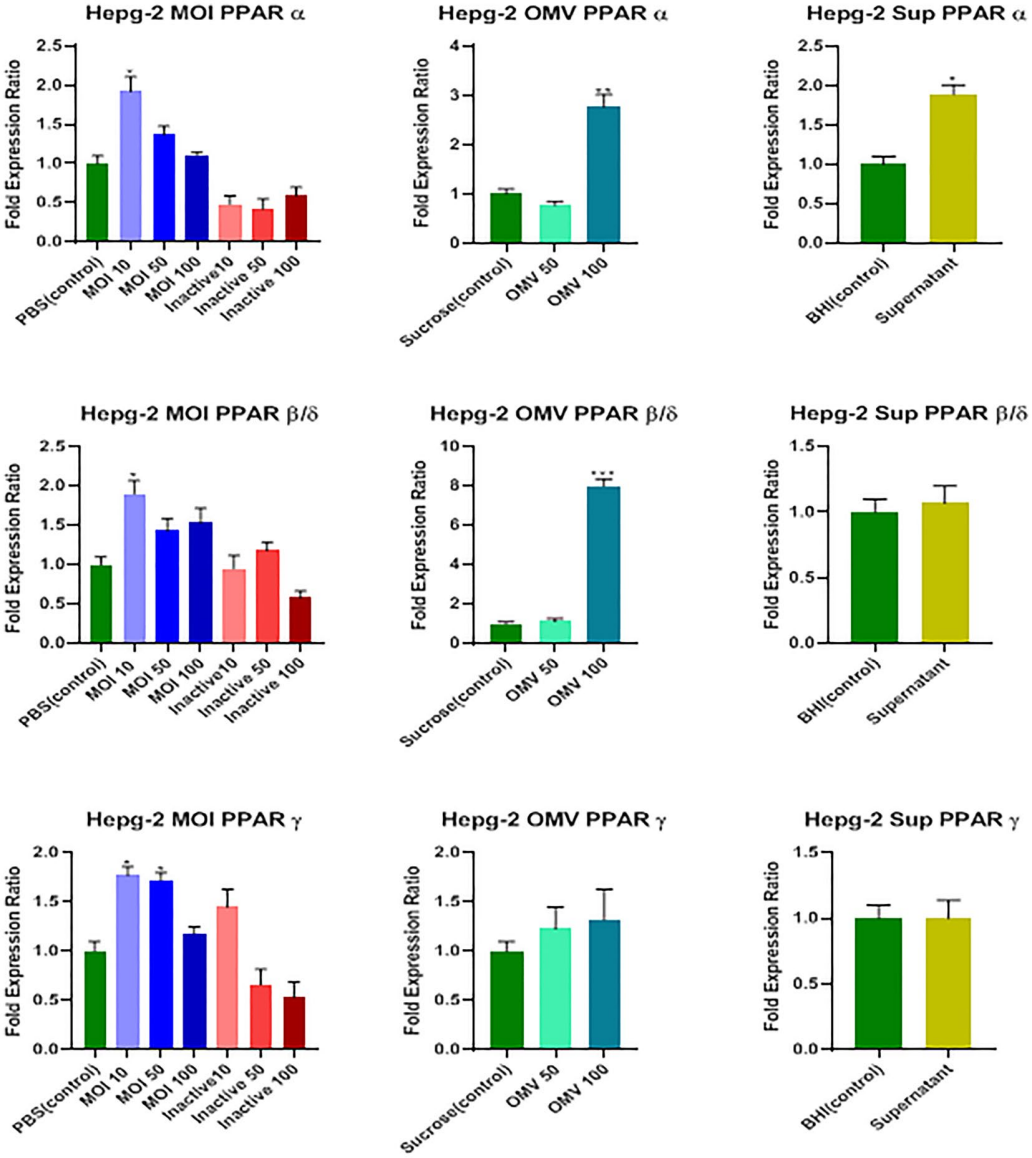
Effects of active and inactive *A.muciniphila* (at MOIs of 10, 50, and 100), its derived OMVs (50 and 100 μg/mL) and 7% (v/v [medium/cell-free supernatant]) cell free supernatant on the expression of PPARS (α, β/δ, and ϒ) genes in HepG-2 cells. Significancy is evaluated in comparison with control. Data are shown as the mean ± SEM. (*) and (^#^) represent significant increase and decrease, respectively. *P < 0.05, **P < 0.01 and***P < 0.001 by one-way ANOVA and t-test statistical analysis. MOI: multiplicity of infection, OMV: outer membrane vesicle, Sup: supernatant.

The MOI 10 of the active form of the bacterium, 100 μg/mL concentrations of OMVs, and 7% (v/v [medium/cell-free supernatant]) concentration of cell-free supernatant noticeably increased the transcriptome level of the *PPARα* gene (*P* = 0.01, *P* = 0.009, and *P* = 0.029, respectively); however, the inactive form of the bacterium did not affect it (*P* > 0.05; Fig. 5).

As shown in Fig. 5, MOI 10 of the active form and 100 μg/mL concentrations of OMVs could remarkably increase the mRNA level of the *PPARβ/δ* gene (*P* = 0.032 and *P* = 0.0005, respectively); nevertheless, neither inactive form nor the cell-free supernatant affected its transcription level (*P* > 0.05).

Finally, there was a significant increase in the mRNA level of the *PPARϒ* gene only at MOI 10 and 50 treatments of the active form of the bacterium (*P* = 0.034 and *P* = 0.047, respectively), and none of the inactive form, OMVs, and cell-free supernatant affected it (*P* > 0.05; Fig. 5).

## Discussion

*A. muciniphila* is an anaerobic Gram-negative bacterium that appropriates about 3–5% of the gut bacteria in healthy humans^12,14^. The evidence has confirmed its role in gut barrier regulation and its involvement in metabolic and homeostatic procedures^4,17^. Recently, a great interest has been attracted on this subject due to its potential of introducing as the next generation probiotics^12^. *A. muciniphila* mainly colonizes in the gut, and due to some leaky gut conditions, the bacterium and related derivatives^52^ might pass through the gut barrier and enter some other organs, such as the liver through the gut-liver axis^4,10,11^. This might happen based on the interaction between the ECS, gut microbiota, and the liver^25^, and the involvement of *PPARs* genes in the regulation of gut barrier permeability^27,40^ and liver metabolism^42–44^. Therefore, this study investigated the effects of *A. muciniphila* and its derivatives on ECS-related and *PPARs* genes in Caco-2 and Hep-G2 cell lines in parallel.

The ECS consists of three main compartments, including cannabinoids, cannabinoid receptors, and metabolic enzymes, concentrated mainly in the brain and some peripheral tissues. They play various roles, such as involvement in the regulation of hunger and satiety, relaxation, protection, immunity, metabolism, decreasing inflammation, and increasing permeability in the GIT. It also influences some diseases, such as obesity, type 2 diabetes, steatosis, fibrogenesis, and alcoholic and nonalcoholic liver diseases, mainly through CB1 function^25–28,53^. Regarding the important role of *A. muciniphila* in gut barrier integrity and decreasing permeability which prevents metabolic disorders associated with obesity^54,55^, the present study showed that MOI 10 of *A. muciniphila* and both concentrations of 50 and 100 μg/mL of *A. muciniphila-*derived OMVs could decrease the level of CB1 mRNA in Caco-2 cells which promotes the idea of using the active *A. muciniphila* as a probiotic candidate and bacterial-derived OMVs for CB1 expression regulation to prevent metabolic disorders associated with obesity.

This study demonstrated that all MOIs (i.e., 10, 50, and 100) of the inactivated form of the bacterium increased the CB1 mRNA level in Caco-2 cells. Consistent with the results of the present study, Everard et al. (2013) observed that in spite of the active bacterium, heat-killed *A. muciniphila* could not improve the thickness of the mucus layer and counteract the fat mass development^56^. Russo et al. (2004) reported that CB1 deficiency might lead to some disorders, such as inflammatory bowel diseases (known as irritable bowel syndrome [IBS]), migraine, fibromyalgia, and psychological disorders^57^. Therefore, the inactivated *A. muciniphila* might be considered a paraprobiotic candidate for the treatment of IBS, especially in conditions that the treatment of live bacteria might exacerbate the inflammation condition.

On the same side, MOI 100 of active bacteria, OMV 50 μg/mL, and cell-free supernatant could decrease the CB2 mRNA level. In contrast, MOI 10 and 50 of inactivated bacteria increased the mRNA level of CB2 significantly. Since the functions of CB1 and CB2 receptors in the gut are in the same direction^26,27,58,59^, the latter results are in line with the present study hypothesis about different effects of active *A. muciniphila,* its derived OMVs, and cell-free supernatant in comparison to those of inactive *A. muciniphila* on ECS function as required. In similar studies on cannabinoid receptors , Rousseaux et al. in 2007 reported that among a diversity of bacteria used in the study (including: *L. acidophilus* NCFM, *L. salivarius* Ls-33, *L. paracasei* Lpc-37, *B. lactis* Bi-07 and *B. lactis* Bl-04, and two *E. coli* strains), only active and heat-inactivated *Lactobacillus acidophilus* NCFM , known as a probiotic, could increase the CB2 mRNA level in HT-29 epithelial cells that made it a considerable candidate for the treament of irritable bowel syndrome and abdominal pains^60^.

In 2016, Scarpelli et al. concluded that the inhibition of FAAH could protect anandamides from degradation leading to more activation of cannabinoid receptors^61^. Therefore, FAAH function has a reverse relationship with cannabinoid receptors. The current study data showed that MOI 50 and 100 of active *A. muciniphila*, both concentrations of OMV 50 and 100 μg/ml, and cell-free supernatant could significantly increase the level of FAAH mRNA in Caco-2 cells.

The MOI 10, 50, and 100 of inactive bacteria also increased FAAH mRNA significantly. Probably, there is a modulatory function of FAAH, since Murakami et el. (2007) discussed that bacterial lipopolysaccharide (LPS) as one of the magic components of MAMPs^19^ in *A. muciniphila*^62^ induces the production of anandamide which is mentioned as a ligand for the CB1 receptor. Meanwhile, the LPS stimulation could not affect FAAH; therefore, it seems natural that the rate of FAAH mRNA increased by more anandamide production to control the overproduction of these ligands^63^. Considerably, there is a reverse relationship in the treatment results of MOI 50 of inactive *A. muciniphila* between CB1 and FAAH in Caco-2 cells.

The PPARs belong to the nuclear receptor superfamily serving as transcription factors that regulate numerous transcriptional activities, such as metabolic, inflammatory, and developmental processes. The PPARs are composed of three isotypes, including PPARα, PPARβ/δ, and PPARϒ, which have different distributions in the human body; however, all three are highly expressed in the colon. The evidence has shown that there is a direct interaction between gut microbiota and PPARs in a way that gut microbiota might induce PPARs expression and activation^40,64^. In 2011, Goto et al. reported that PPARα stimulates fatty acid oxidation in adipocytes and ameliorates metabolic disorders^65^. In 2003, prior to this study, Wang et al. declared that PPARβ/δ prevents obesity through fat metabolism and energy expenditure^66^. La Cour Poulsen et al. in 2012 explained that numerous types of fatty acids and their derivatives could activate PPARs. Furthermore, it has been suggested that there is a relationship between the microbiota, fatty acid metabolism, and PPARs.

In 2019, Ashrafian et al. reported that *A. muciniphila* and its derived OMVs stimulated fatty acid oxidation and energy metabolism. These processes occurred in addition to the increased expression of PPARα and PPARϒ^49^. Recently, in 2020, Wang et al. reviewed that *A. muciniphila* produces short-chain fatty acids (SCFAs), which affect glucose and lipid homeostasis. It also increases fatty acid oxidation in the intestine and adipose tissue^54,67,68^. Consistent with these findings, we observed that MOIs 50 and 100 of the active form, MOIs 10 and 50 of the inactivated form, both 50 and 100 μg/mL concentrations of OMVs, and cell-free supernatant could significantly increase the rate of PPARα transcriptome. The PPARβ mRNA level was almost increased by the same treatments significantly by the effects of MOI 50 of the active and MOI 10 of inactivated forms and OMV 100 μg/mL. The result received for PPARϒ was in the same direction as previous isotypes in such a way that mRNA level was increased significantly by MOIs 50 and 100 of the active and MOI 10 of the inactivated forms and both 50 and 100 μg/mL concentrations of OMVs. All these results confirmed the positive effects of *A. muciniphila* and related derivatives on the transcription of *PPARs* genes, probably through affecting fatty acids oxidation and energy metabolism.

In gut microbiota overgrowth conditions or tight junctions’ impairments, the integrity of the gut decreases, which leads to increasing the gut permeability and translocation of bacteria from the gut lumen to the portal and/or systemic circulation. In such conditions, the bacteria might enter the liver through the gut-liver axis^11^. In this study, MOI 50 of active *A. muciniphila* decreased the mRNA level of the CB1 receptor significantly in Hep-G2 cell lines; nevertheless, inactivated *A. muciniphila*, OMVs, and cell-free supernatant could not affect CB1 receptor transcription. Active *A. muciniphila* induces beneficial effects on hepatocytes through the downregulation of the CB1 receptor since Mallat et al. in 2013 explained that the CB1 receptor is expressed in hepatocytes and hepatic myofibroblasts and involved in numerous liver diseases, such as alcohol-induced liver disease, nonalcoholic fatty liver disease, fibrogenesis, and cardiovascular alterations associated with cirrhosis^43^.

Studies have shown that the expression of CB2 receptors in hepatocytes is modest, mainly contributes to hepatoprotective, anti-inflammatory, antioxidant, and immunomodulatory effects^69,70^, and plays a prohibiting role in liver fibrosis and alcohol-induced liver damage, compared to CB1 receptor^28,43,71^. In this study, neither active nor inactivated *A. muciniphila* could affect CB2 receptor expression; however, 100 μg/mL concentration of OMVs and cell-free supernatant noticeably increased the CB2 expression in mRNA level representing the more influence of the bacterium derivatives and metabolites rather than the bacterium itself. These results might have the root in *A. muciniphila-*derived OMVs and related cell-free supernatant that might preserve as therapeutic agents to improve liver health conditions. Moreover, it was observed that none of the treatments had an influence on FAAH expression except 100 μg/mL concentration of OMVs. This finding might be considered satisfactory since FAAH is a hydrolyzing enzyme for the ligands of both receptors and naturally balances the ECS-dependent health of the liver.

*A. muciniphila* produces SCFAs, such as propionate, butyrate, and acetate^54^ which can activate PPAR *α* expression through which prevents lipid accumulation in the liver^72^. The PPARα is mostly found in hepatocytes, plays a critical role in fatty acid uptake and fatty acid oxidation, decreases the production of very-low-density lipoprotein, and increases high-density lipoprotein. It also downregulates the hepatic inflammatory processes^32^. In the current study, MOI 10 of the active *A. muciniphila*, 100 μg/mL concentration of OMVs, and the-cell free supernatant considerably increased the PPARα expression in mRNA level. This result suggests that less amount of the active bacterium might promote PPARα expression better than higher amounts which might cause no inflammation in liver tissues. Similar results have been obtained for *PPARβ/δ* as it was observed that MOI 10 of the active *A. muciniphila* and 100 μg/mL concentration of OMVs significantly increased the mRNA level of *PPARβ/δ*.

Previous studies revealed that the potential role of *PPARβ/δ* in hepatocytes is apparent; they assumed that this gene is highly expressed in these cells and regulates glucose utilization and fatty acid metabolism. It also participates in the alleviation of inflammation and fibrosis^32,72,73^. Transcription analysis showed that *A. muciniphila’s* MOIs of 10 and 50 could enhance the PPARϒ transcriptome level, which is according to the results of studies of several researchers who concluded that gut microbiota is associated with metabolism, including PPARs expression related pathways^72,74^. Wagnerberger et al. in 2013 reported that intake of *Lactobacillus casei* upregulated hepatic PPARϒ leading to inhibition of Toll-like receptor 4 and suppression of steatosis^75^. Additionally, in previous studies in 2011, Nan et al. declared that the overexpression of PPARϒ can influence some liver diseases, such as reducing effect on steatosis, inflammation, and fibrosis in steatohepatitis murine model^76^. In a recent study, Keshavarz et al. reported that MOI 10 of heat-killed bacteria increased the expression of PPARϒ remarkably^18^. The effect of PPARϒ on fatty acid oxidation and glucose homeostasis as an insulin sensitizer was also lately reviewed by Wu et al.^72^.

In normal conditions, the bacterial derivatives might pass through the gut barrier either directly or via dynamin-dependent endocytosis and then translocate toward the liver^52^. In some leaky gut conditions, the bacteria might translocate from the gut to the liver through the gut-liver axis^10,11^. Some associations might be observed in the gene transcription of the cells in both organs. For example, as shown in Figs. 2 and 4, active *A. muciniphila* could decrease the level of *CB1* transcription in both cell lines. Furthermore, OMVs treatments in both cell lines increased the *FAAH* mRNA level. The transcription of *PPARα, β/δ*, and *ϒ* was increased by active *A. muciniphila* in both cell lines (Figs. 3 and 5). The increased level of *PPARα* by the treatment of inactive bacteria and derived OMVs in Caco-2 cells was in line with HepG-2 cells (Figs. 3 and 5). A similar result was observed by the effect of OMVs treatments on PPAR*β/δ* transcription in two cell lines (Figs. 3 and 5). One contradiction was the effects of OMVs and cell-free supernatant treatments on the CB2 transcription level, which were contradictory in two cell lines. They might affect *CB2* gene transcription with different mechanisms in the aforementioned cell lines, which requires more investigation.

The limitations of the current study were accomplishing these experiments in expression level and performing the same procedure under in vivo conditions, especially to investigate the correlation of gut and liver gene expressions affected by the aforementioned treatments.

In conclusion, we considered the positive effects of *A. muciniphila* and its derivatives, such as OMVs and bacterial metabolites, on controlling the activity of ECS compartments which might influence obesity, metabolic disorders, and liver diseases depending on their type. According to the present study results, *A. muciniphila* and its derivatives might be considered probiotic, paraprobiotic, and postbiotic candidates to protect organs against metabolic syndromes and liver diseases.

It is worth mentioning that there is an interaction between eCBs and *PPARs* genes which introduces some possible pathways of PPARs activation by eCBs either directly or indirectly^41^. Considering the essential roles of PPARs as nuclear receptors in the regulation of energy homeostasis, metabolism, cell differentiation, and inflammation^77–80^, the evidence suggests that numerous ECB functions, such as analgesic, neuroprotective, neuronal function modulation, anti-inflammatory, metabolic, anti-tumor, gastrointestinal, and cardiovascular effects of some cannabinoids are mediated by PPARs^41^. This association attracts the attention to scrutinize all possible pathways that might influence this study’s findings.

## Supplementary Information

Supplementary Information.

## Data Availability

All data that support all the experimental findings in this article is available in the Supplementary Data File provided.
