# Author Correction: Effects of active, inactive, and derivatives of *Akkermansia muciniphila* on the expression of the endocannabinoid system and *PPARs* genes

**DOI:** 10.1038/s41598-022-16591-8

**Published:** 2022-07-19

**Authors:** Farinaz Ghaderi, Fattah Sotoodehnejadnematalahi, Zahra Hajebrahimi, Abolfazl Fateh, Seyed Davar Siadat

**Affiliations:** 1 Department of Biology, Science and Research Branch, Islamic Azad University, Tehran, Iran; 2 A&S Research Institute, Ministry of Science Research and Technology, Tehran, Iran; 3 Department of Mycobacteriology and Pulmonary Research and Microbiology Research Center (MRC), Pasteur Institute of Iran, Tehran, Iran; 4 Microbiology Research Center (MRC), Pasteur Institute of Iran, Tehran, Iran

Correction to: *Scientific Reports* https://doi.org/10.1038/s41598-022-13840-8, published online 15 June 2022

In the original version of this Article, Fateh Abolfazl was incorrectly listed as a corresponding author. The correct corresponding author for this Article is Seyed Davar Siadat. Correspondence and request for materials should only be addressed to d.siadat@gmail.com.

The original Article has been corrected.

