# Supplementary material for: Effects of active, inactive, and derivatives of Akkermansia muciniphila on the expression of the endocannabinoid system and PPARs genes
Source: Sci Rep. 2022 Jun 15;12:10031. doi: 10.1038/s41598-022-13840-8 (PMC9200819; doi:10.1038/s41598-022-13840-8)
Supplement: Supplementary file 1 — Supplementary Information. [file 41598_2022_13840_MOESM1_ESM.docx]

**Supplementary table 1.**The sequence of primers for confirmation of *A.muciniphila*

| Primer Name | Forward Primer | Reverse Primer | Product Size | Annealing Tm | Reference |
| --- | --- | --- | --- | --- | --- |
| *A.muciniphila* 16s rRNA | CAGCACGTGAAGGTGGGGAC | CCTTGCGGTTGGCTTCAGAT | 316 | 59°c | 1 |

**Supplementary table 2.** Specifications of Primers Used in RT-qPCRs

| Primer Name | Forward Primer | Reverse Primer | Product Size  (bp) | Annealing Tm  (°c) | GeneBank No. | Reference |
| --- | --- | --- | --- | --- | --- | --- |
| *GAPDH*  *CBR1*  *CBR2*  *FAAH*  *PPAR α*  *PPAR β/δ*  *PPAR ϒ* | AACAGCCTCAAGATCATCAGCAA  GATGTCTTTGGGAAGATGAACAAGC  GCTATCCACCTTCCTACAAAGC  GTGGTGCTGACCCCCATGCTGG  CGGTGACTTATCCTGTGGTCC  ACTGAGTTCGCCAAGAGCATCGGGATCAGCTCCGTGGATCT | GATGGCATGGACTGTGGTCAT  AGACGTGTCTGTGGACACAGACATGG  CTCAGCAGGTAGTCATTGGGG  TCCACCTCCCGCATGAACCGCAGACA  CCGCAGATTCTACATTCGATGTT  ACGCCATACTTGAGAAGGGTAATGCACTTTGGTACTCTTGAAGTT | 120  309  172  302  79  77  186 | 55  64  55  60  55  55  55 | 001256799  001365874  001841  001441  001393947  001171819  001354666 | ^2^  ^3^  ^4^  ^5^  ^6^  ^7^  ^8^ |

**References**

1 Zhai, R. *et al.* Strain-specific anti-inflammatory properties of two Akkermansia muciniphila strains on chronic colitis in mice. *Frontiers in cellular and infection microbiology* **9**, 239 (2019).

2 Ebnerasuly, F., Hajebrahimi, Z., Tabaie, S. M. & Darbouy, M. Effect of simulated microgravity conditions on differentiation of adipose derived stem cells towards fibroblasts using connective tissue growth factor. *Iranian journal of biotechnology* **15**, 241 (2017).

3 Sarnataro, D. *et al.* Plasma membrane and lysosomal localization of CB1 cannabinoid receptor are dependent on lipid rafts and regulated by anandamide in human breast cancer cells. *FEBS letters* **579**, 6343-6349 (2005).

4 Aragão, L. G. H. *et al.* A cannabinoid receptor agonist shows anti-inflammatory and survival properties in human SARS-CoV-2-infected iPSC-derived cardiomyocytes. *bioRxiv* (2021).

5 Ligresti, A. *et al.* Possible endocannabinoid control of colorectal cancer growth. *Gastroenterology* **125**, 677-687 (2003).

6 Guan, C.-Y. *et al.* Down-Regulated miR-21 in Gestational Diabetes Mellitus Placenta Induces PPAR-α to Inhibit Cell Proliferation and Infiltration. *Diabetes, Metabolic Syndrome and Obesity: Targets and Therapy* **13**, 3009 (2020).

7 Wyckelsma, V. L. *et al.* Vitamin c and e treatment blunts sprint interval training–induced changes in inflammatory mediator-, calcium-, and mitochondria-related signaling in recreationally active elderly humans. *Antioxidants* **9**, 879 (2020).

8 Maeba, R. & Nakahara, S.-i. Extrinsic effectors regulating genes for plasmalogen biosynthetic enzymes in HepG2 cells. *Biomed Res Clin Pract* **2** (2017).
